# Supplementary material for: Differential regulation of the proteome and phosphoproteome along the dorso-ventral axis of the early Drosophila embryo
Source: eLife. 2024 Sep 2;13:e99263. doi: 10.7554/eLife.99263 (PMC11466282; doi:10.7554/eLife.99263)
Supplement: Supplementary file 1. [file elife-99263-supp1.docx]

| **Phenotypic Class** | **marker gene ->** | **dpp** | **sog** | **snail** |
| --- | --- | --- | --- | --- |
|  | **genotype** |  |  |  |
| dorsalised | *gd^9^* | +++ | - | - |
| lateralized | *tl^rm10^/tl^rm9^* | *-* | +++ | - |
| ventralised | *spn27a^ex^/*  *Df(2L)6374* | *-* | +++ | +++ |
| ventralised | *Tl^10B^ /*  *Df(3R)ro80b* | *-* | around head fold | +++  (gap around head fold) |

**Table 1:**

Genotypes of mutants used for mass spectrometry and expression of diagnostic zygotic genes in the mutant embryos.

Legend: +++: entire circumference at 50% egg length; *-*: expression only at the poles, no expression in the trunk region; - : no expression.
